# Movable Kidneys

**Published:** 1911-05-06

**Authors:** 


### Surgery.

### MOVABLE KIDNEYS.

All kidneys move somewhat with respiration. Many have a wide range of movement without ever causing their owners any trouble: others again, with merely a moderate range of movement, are associated with symptoms of pain and discomfort that may or may not be due to this mobility. Yet again an undue mobility of the kidney may be the cause of great pain and severe illness.

Seeing that all degrees of seriousness may be associated with a similar lesion, the practitioner must have considerable difficulty in deciding whether to advise a patient to have his kidney sewn up or to steer clear of a surgeon. Many people have submitted to the operation of nephropexy and have only been disappointed, while others have obtained complete relief from it.

The cases may be divided into those who are only incommoded in an active life and those who at periodical intervals are quite incapacitated.

The former suffer with a dull ache in the back and side (nearly always the right); the ache relieved by lying down, but which quickly returns when the patients are up and about. It does not confine them to bed, but prevents them doing a whole day's work, or getting much enjoyment out of life; their whole horizon is grey with the presence of the ache or by the thought of its return when it is absent. When such a condition can be proved to be due to an increased mobility of the kidney and nothing else, the operation of nephropexy will probably cure it. Unfortunately, the number of conditions that will produce these symptoms is legion, while the number of cases where the kidney is the cause is small, and the number where it is the only cause still smaller. As a result, this operation has of late years fallen somewhat into disrepute.

What, then, are the conditions that may be present with an unusual range of movement of the kidney and the symptoms mentioned above? Firstly, the patient may suffer from chronic constipation, and the distended cæcum may cause a dull ache in the loin and side. The cæcum can in these cases be palpated and be found distended in the iliac fossa, and the patient will wince when this is pressed upon. If palpation of the cæcum causes pain, and of the kidney does not, then an operation for stitching up the kidney will do no good, however freely mobile that organ may be. If palpation of both organs gives rise to pain, then operation on the kidney will probably be very little use in relieving the symptoms.

Again, the kidney may be a part of a general visceroptosis. Operation on one or both kidneys may result in keeping them in place, but unless the liver, the stomach, and all the other abdominal viscera are also stitched up into position, it is not likely to relieve them of their pain.

In young girls the same symptoms may result from a general weakness of all the muscles and ligaments of the spinal column. They may have freely mobile kidneys, but they also have some degree of lateral curvature, associated with other static deformities, such as flat-foot.

When all these cases have been excluded there remain a few where the mobile kidney does cause the pain and where operation will do good. Where both kidneys are freely movable much hope must not be put forward of relief; when one kidney only is involved the greater the range of movement the more likely is operation to be of benefit. The kidney can generally be brought down into the iliac fossa on deep inspiration and held there between the fingers of the two hands; it is also tender to the touch. If the range of movement is so small that the thickest part of the kidney only can be gripped, and if such a grip does not cause pain, then it is doubtful whether operation is going to be of much benefit. There are such people as neurotics; it is so easy to label them and so difficult to treat them; but if one be told that he or she has a movable kidney, he will not rest until that has been sewn up, when the pain will return until he is told the appendix or the colon or some other organ is at fault.

In the other class of movable kidneys more marked symptoms are present: the patient is suddenly taken with acute pain in the loin, which radiates down the thighs, and this is associated with vomiting, and there is tenderness of the abdominal wall in this region; distension of the intestines with gas ensues. These attacks are given the name of Dietl's crises. It will be seen that such must be diagnosed firstly from other acute abdominal lesions, and when the kidney is selected as the site of the lesion two other renal conditions which arise in a similar way must be excluded—attacks of renal colic due to stone, and an acute pyelonephritis, which is commonest in pregnant women, but may occur in them without pregnancy or may be present in a man. An *x*-ray examination will give evidence of stone; a bacteriological examination of the urine will disclose micro-organisms. The vomiting in these cases is said to be due to the kidney dragging down the duodenum and pylorus; in some cases jaundice has been observed from the resulting kinking of the bile-ducts.

In other cases hæmaturia may result from obstruction to the blood in the vessels of the hilum of a movable kidney. These are however rare, and it is important to bear in mind that hæmaturia may be the first symptom of renal tuberculosis; no case of hæmaturia, therefore, should be put down to mobility of the kidney until tubercle has been carefully excluded as well as stone.

Lastly, mobility of the kidney may give rise to hydronephrosis; a vicious cycle is then set up. The kinked ureter causes the obstruction, the enlarged kidney causes the kidney to drop still further, and the ureteric kink becomes more complete and so gives rise to further distension.

In this last series of cases operation may be confidently urged, with the promise of relief from the trouble when once the mobile kidney has been diagnosed as the cause.

